# Obstructive sleep apnea syndrome in children with cerebral palsy in Brazil: a multicenter study

**DOI:** 10.1016/j.jped.2025.101432

**Published:** 2025-08-22

**Authors:** Bruno Leonardo Scofano Dias, Fernanda Marinho de Lima, Daniela Fava, Fernanda Jordão Pinto Marques, Frederico José de Carvalho Godinho, Alessandra Lemos de Carvalho, Sara Virgínia Paiva Santos, Fernanda de Lourdes da Cunha Pinto, Danielle Bruno Jardim, Wilerson Marques Bessa

**Affiliations:** Rede SARAH de Hospitais de Reabilitação, Brazil

**Keywords:** Sleep apnea, Obstructive, Cerebral palsy, Children, Risk, Questionnaire

## Abstract

**Objectives:**

To evaluate the prevalence of high risk for obstructive sleep apnea syndrome (HR-OSAS) in Brazilian children with cerebral palsy (CP) using the Pediatric Obstructive Sleep Apnea Screening Tool (PosaST) and to analyze its association with demographic, clinical and functional (Gross Motor Function Classification System [GMFCS]) variables.

**Methods:**

Multicenter, cross-sectional, exploratory study.

**Results:**

There were 312 children (median age 6.0 years, IQR 5.0–8.0) included. The prevalence of HR-OSAS in GMFCS I-V was 9.0 %. The prevalence of HR-OSAS in GMFCS V (14.7 %) was significantly higher compared to GMFCS I-IV (6.4 %) and to the frequency of OSAS in typically developing (TD) children assessed by polysomnography (5.8 %) according to literature data. Significantly higher frequencies of palatine tonsil hypertrophy, hospitalizations and outpatient antibiotic use for respiratory causes (last 12 months), gastroesophageal reflux disease, drooling and epilepsy were found in GMFCS V. Palatine tonsil hypertrophy was significantly associated with HR-OSAS. GMFCS V was significantly correlated with HR-OSAS at the expense of its significantly higher prevalence of palatine tonsil hypertrophy.

**Conclusions:**

The prevalence of HR-OSAS in Brazilian children with CP (GMFCS V) was higher than the frequency of OSAS in TD children assessed by polysomnography. HR-OSAS was significantly more prevalent in GMFCS V compared with GMFCS I-IV. Palatine tonsil hypertrophy was significantly associated with HR-OSAS. GMFCS V was significantly correlated with HR-OSAS due to its significantly higher prevalence of palatine tonsil hypertrophy. PosaST may be a reliable questionnaire for Brazilian children with CP, but studies are needed to define the HR-OSAS cutoff score in this population.

## Introduction

Sleep-disordered breathing (SDB) is a group of respiratory disorders observed during sleep, with higher prevalences in preschool and school children[1]. Its main risk factor is adenotonsillar hypertrophy[1,2]. Obstructive sleep apnea syndrome (OSAS) is an SDB subtype. It is defined as a syndrome of upper airway dysfunction during sleep, characterized by snoring and/or increased respiratory effort secondary to increased upper airway resistance[3]. OSAS affects up to 5.8 % of typically developed (TD) children assessed by polysomnography [4, 5, 6] and 10.5 % of those evaluated by questionnaires[7]. Most children are approximately 2 to 8 years of age, due to the relative size of the upper airway lymphatic tissue[3]. The most predictive symptoms of OSAS are snoring, respiratory distress during sleep, and parentally witnessed apnea[8]. OSAS confers a risk of developing associated behavioral, learning, metabolic and cardiovascular morbidities[2,9,10,11].

Cerebral palsy (CP) is the most common physical disability in childhood[12]. Children with CP are at increased risk for OSAS [13, 14, 15]. In addition to adenotonsillar hypertrophy, children with CP may also present other characteristics that result in airway collapse or obstruction, such as hypotonia of the palate and constrictor muscles, glossoptosis, and laryngeal dystonia [16].

Nocturnal in-laboratory polysomnography is the gold standard test for the diagnosis of OSAS in children with clinical manifestations of SDB [1]. However, in countries where access to polysomnography is poor, assessments from questionnaires are effective clinical tools used to identify patients at increased risk for OSAS [1,17,18]. Some American [5,19,20], European [14,16,21] and Asian [22] studies used questionnaires for assessment and demonstrated higher prevalences of OSAS in children with CP compared to controls.

The Pediatric Obstructive Sleep Apnea Screening Tool (PosaST) questionnaire consists of six questions about pediatric sleep and has high sensitivity and moderate specificity for high risk for moderate to severe OSAS (HR-OSAS) in preschoolers and schoolchildren [1].

This study aimed to: (1) evaluate Brazilian children with CP consecutively treated at the SARAH Network of Rehabilitation Hospitals, regarding the prevalence of HR-OSAS using the PosaST Brazilian version; (2) analyze its association with demographic, clinical and functional variables.

The hypotheses were: (1) the prevalence of HR-OSAS would be significantly higher in children with CP compared to TD children assessed by polysomnography and questionnaire; (2) children with CP with more severe functional impairment would have a significantly higher prevalence of HR-OSAS than those less impaired.

## Method

### *Setting*

This study was performed at eight units of the SARAH Network of Rehabilitation Hospitals, a public network of rehabilitation hospitals in Brazil, present in four of the five regions of the country. Some of the units serve both inpatients and outpatients, and others only outpatients. In all units, there were one or two professionals responsible for collecting data, with one coordinating pediatrician integrating all units and processing the data.

### *Design and participants*

The study used a multicenter, cross-sectional and exploratory design. There are no reliable studies regarding the prevalence of CP in Brazilian children. Thus, it was not possible to carry out the sample size calculation, and a convenience sample was used. Participants were recruited from a sample of children with CP consecutively treated at the SARAH Network of Rehabilitation Hospitals between June and November 2024. Inclusion criteria were children with CP aged 3 to 9 years. Exclusion criteria were a history of adenotonsillectomy, continuous use of inhaled corticosteroids, use of tracheostomy tubes and/or oxygen supplementation. The study was approved by the Research Ethics Committee of the SARAH Network of Rehabilitation Hospitals (Certificate of Submission for Ethics Assessment 77,935,124.4.0000.0022).

### *Data collection, measures and variables*

Data were collected by pediatricians and one speech therapist. Patients’ legal guardians were approached, the objectives of the study were explained, and informed consent for the research and publication of results was completed. Demographic and functional data were collected from the patient’s electronic medical records. Clinical data were accessed through anamnesis and physical examination. The PosaST was applied in the form of an interview, lasting approximately ten minutes, using a standardized technique. Online meetings were held to standardize the method of applying the scale and collecting data. Three hundred and nineteen children met the inclusion criteria.

Functional skills were analyzed using the Brazilian version of the Gross Motor Classification System (GMFCS) [23], a five increasing severity levels (I to V), globally used scale. Friedman criteria (zero - tonsils not visualized; 1 - tonsils visible in the tonsillar fossa; 2 - tonsils visible beyond the anterior pillars; 3 - tonsils occupying ¾ of the space between the anterior pillars; 4 - tonsils completely obstructing the airway between the anterior pillars) [3] were used to access palatine tonsil volumes. Prevalence of OSAS in TD children was defined as those assessed by polysomnography^6^ and questionnaire (Pediatric Sleep Questionnaire - PSQ)[7].

The PosaST is translated and validated for Brazilian Portuguese and culture in children aged 3 to 9 years, with acceptable validity and good reliability and agreement with the apnea-hypopnea index (AIH - number of apneas and hypopneas per hour of total sleep time on polysomnography). A score ≥ 2.72 is considered indicative of HR-OSAS. All questions are answered using the Likert scale: ''never'' (0), ''rarely'' (once a week; 1), ''occasionally'' (twice a week; 2), ''frequently'' (three to four times a week; 3) and ''almost always'' (more than four times a week; 4). The authors of the scale validation in Brazil authorized its use in this study^1^.

### *Statistical analysis*

The data were described using median (interquartile range - IQR) or mean (standard deviation - SD) for continuous variables and frequency (percentage) for categorical variables. Categorical variables were presented as follows: age (3-9/6-9 years); sex (male/female); Friedman criteria (0; 1; 2; 3; 4) and (0-2/3-4); hospitalizations for respiratory causes in the last 12 months (yes/no), (more than one, yes/no) and (1-2; 3-4; 5-6; > 6); respiratory complications with antibiotics used in the last 12 months (more than one, yes/no) and (1-2; 3-4; 5-6; > 6); gastroesophageal reflux disease (GERD) (yes/no); drooling (yes/no); epilepsy (yes/no); GMFCS (I; II; III; IV; V) and (I-IV/V); PosaST (OSAS low/high risk).

Univariate models were constructed for the following comparisons: prevalences of demographic and clinical variables in GMFCS I-IV vs GMFCS V samples; the prevalence of HR-OSAS in GMFCS I-V and GMFCS V samples vs prevalence of OSAS in TD children assessed by polysomnography and questionnaire. Multivariate binary logistic regression models for HR-OSAS and Friedman criteria levels 3-4 were constructed. Bilateral p-values less than or equal to 0.05 were considered significant. Estimates were presented with a 95% confidence interval (95 % CI) and the odds ratio (OR) was calculated.

Analyses were performed in Jamovi 2.3.16 (Jamovi, 2022). STROBE reporting guidelines were followed.

## Results

Among the 319 children who met the inclusion criteria, seven were removed after applying the exclusion criteria. The final sample consisted of 312 children (GMFCV I-V). The continuous variable age did not present a normal distribution according to the Shapiro-Wilk test. Most of the 312 children (59.0 %) were aged 6-9 years (median 6.0 years, IQR 5.0-–8.0), 164 males, 69.5 % GMFCS I-IV, from the five regions and 14 of the 26 states of the country (Table 1). The GMFCS I-IV sample consisted of 217 children, 60.4 % aged 6-9 years (median 6.0 years, IQR 4.0-–8.0), 111 males. The GMFCS V sample comprised 95 children, 55.8 % aged 6-9 years (median 6.0 years, IQR 4.0-–8.0), and 54 males. There were no significant differences in age groups and sex frequencies comparing GMFCS I-IV vs GMFCS V samples (Table 2).

**Table 1:** Frequencies of demographic, clinical and functional characteristics.

	**GMFCS I-V**
	*n* = 312
**Characteristic**	n ( %)
**Age (years)**	
3 - 5	128 (41.0)
6 - 9	184 (59.0)
**Sex**	
Female	147 (47.1)
Male	165 (52.9)
**Friedman classification**	
0	36 (11.5)
1	98 (31.4)
2	105 (33.7)
3	69 (22.1)
4	4 (1.3)
**Friedman classification**	
0 - 2	239 (76.6)
3 - 4	73 (23.4)
**Hospitalizations for respiratory causes (last 12 months)**	
0	264 (84.6)
1 - 2	35 (11.2)
3 - 4	8 (2.6)
5 - 6	3 (1.0)
> 6	2 (0.6)
**Hospitalizations for respiratory causes (last 12 months)**	
Yes	48 (15.4)
No	264 (84.6)
**Hospitalizations for respiratory causes (> 1 in the last 12 months)**	
Yes	13 (4.2)
No	299 (95.8)
**Outpatient use of antibiotics (last 12 months)**	
0	129 (41.3)
1 - 2	96 (30.8)
3 - 4	50 (16.0)
5 - 6	10 (3.2)
> 6	27 (8.7)
**Outpatient use of antibiotics (last 12 months)**	
Yes	183 (58.7)
No	129 (41.3)
**Outpatient use of antibiotics (> 1 in the last 12 months)**	
Yes	87 (27.9)
No	225 (72.1)
**GERD**	
Yes	36 (11.5)
No	276 (88.5)
**Drooling**	
Yes	136 (43.6)
No	176 (56.4)
**Epilepsy**	
Yes	138 (44.2)
No	174 (55.8)
**GMFCS**	
I	85 (27.2)
II	35 (11.2)
III	45 (14.4)
IV	52 (16.7)
V	95 (30.5)
**GMFCS**	
I-IV	217 (69.5)
V	95 (30.5)
**HR-OSAS**	
Yes	28 (9.0)
No	284 (91.0)

GERD, gastroesophageal reflux disease; GMFCS, Gross Motor Function Classification System; HR-OSAS, high risk for obstructive sleep apnea syndrome.

**Table 2:** Frequencies and univariate models for comparison between prevalence of demographic, clinical and functional characteristics.

**Characteristic**	**GMFCS I-IV**	**GMFCS V**	p*	**CI 95%**	**OR**
	*n* = 217	*n* = 95			
	n ( %)	n ( %)			
**Age (years)**					
3 - 5	86 (39.6)	42 (44.2)	0.45	−0.68, 0.30	0.83
6 - 9	131 (60.4)	53 (55.8)			
**Sex**					
Female	106 (48.8)	41 (43.2)	0.35	−0.26, 0.71	1.26
Male	111 (51.2)	54 (56.8)			
**Friedman classification**					
0 - 2	179 (82.5)	60 (63.2)	**<0.001**	0.47, 1.57	2.75
3 - 4	38 (17.5)	35 (36.8)			
**Hospitalizations for respiratory causes (last 12 months)**					
Yes	25 (11.5)	23 (31.9)	**0.005**	0.27, 1.53	2.45
No	192 (88.5)	72 (68.1)			
**Hospitalizations for respiratory causes (> 1 in the last 12 months)**					
Yes	5 (2.3)	8 (8.4)	**0.02**	0.22, 2.51	3.89
No	212 (97.7)	87 (91.6)			
**Outpatient use of antibiotics (last 12 months)**					
Yes	118 (54.4)	30 (31.6)	**0.02**	0.09, 1.11	1.82
No	99 (45.6)	65 (68.4)			
**Outpatient use of antibiotics (> 1 in the last 12 months)**					
Yes	62 (28.6)	25 (22.1)	0.68	−0.66, 0.43	0.89
No	155 (71.4)	70 (77.9)			
**GERD**					
Yes	15 (7.0)	21 (19.2)	**<0.001**	−2.05, −0.63	0.26
No	202 (93.0)	74 (80.8)			
**Drooling**					
Yes	59 (27.2)	77 (81.0)	**<0.001**	−3.03, −1.84	0.09
No	158 (72.8)	18 (19.0)			
**Epilepsy**					
Yes	67 (30.9)	71 (74.7)	**<0.001**	1.35, 2.44	6.62
No	150 (69.1)	24 (25.3)			
**HR-OSAS**					
Yes	14 (6.4)	14 (14.7)	**0.02**	0.13, 1.70	2.51
No	203 (93.6)	81 (85.3)			

*Chi-square.

CI, confidence interval; GERD, gastroesophageal reflux disease; GMFCS, Gross Motor Function Classification System; HR-OSAS, high risk for obstructive sleep apnea syndrome; OR, odds ratio.

The following variables were significantly more frequent in the GMFCS V sample compared to GMFCS I-IV: Friedman criteria 3–4 (OR = 2.75; 95 % CI 0.47, 1.57); hospitalizations for respiratory causes in the last 12 months (OR = 2.45; 95 % CI 0.27, 1.53); more than one hospitalization for respiratory causes in the last 12 months (OR = 3.89; 95 % CI 0.22, 2.51); use of antibiotics in the last 12 months (OR = 1.82; 95 % CI 0.09, 1.11); GERD (OR = 0.26; 95 % CI −2.05, −0.63); drooling (OR = 0.09; 95 % CI −3.03, −1.84); epilepsy (OR = 6.62; 95 % CI 1.35, 2.44); HR-OSAS (OR = 2.51; 95 % CI 0.13, 1.70) (Table 2).

There was a significantly higher prevalence of HR-OSAS in the GMFCS V sample (14.7 %) compared to the prevalence of OSAS in TD children assessed by polysomnography (OR = 0.01; 95 % CI −7.79, −1.86). The comparisons between the prevalence of HR-OSAS in GMFCS I-V and TD children assessed by polysomnography (OR = 1.59; 95 % CI 0.95, 2.67) and PSQ (OR = 0.84; 95 % CI 0.55, 1.28) and between GMFCS V and TD children evaluated by PSQ (OR = 1.48; 95 % CI 0.82, 2.66) showed no significant differences (Table 3).

**Table 3:** Univariate models for comparison between prevalences of high risk for obstructive sleep apnea syndrome in children with CP and of obstructive sleep apnea syndrome in typically developed children.

**Sample**	**Prevalence**	p*	**CI 95%**	**OR**
**GMFCS I-V**	9.0 %	0.422	0.55, 1.28	0.84
**PSQ^7^**	10.5 %			
**GMFCS I-V**	9.0 %	0.072	0.95, 2.67	1.59
**PSG^6^**	5.8 %			
**GMFCS V**	14.7 %	0.191	0.82, 2.66	1.48
**PSQ^7^**	10.5 %			
**GMFCS V**	14.7 %	**0.001**	1.45, 5.41	2.80
**PSG^6^**	5.8 %			

*Chi-square.

CI, confidence interval; GMFCS, Gross Motor Function Classification System; OR, odds ratio; PSG, polysomnography; PSQ, Pediatric Sleep Questionnaire.

The multivariate model for HR-OSAS in GMFCS I-V excluding the Friedman criteria as a dependent variable showed significant positive associations with more than one period of antibiotics use for respiratory causes in the last 12 months (OR = 5.54; 95 % CI 0.68, 2.75) and GMFCS V (OR = 3.15; 95 % CI 0.24, 2.05). The multivariate model for HR-OSAS in GMFCS I-V including the Friedman criteria as a dependent variable showed significant positive correlations with Friedman criteria 3–4 (OR = 8.64; 95 % CI 1.18, 3.13) and more than one period of antibiotics use for respiratory causes in the last 12 months (OR = 4.86; 95 % CI 0.45, 2.72). The multivariate model for Friedman criteria 3–4 showed significant positive associations with GMFCS V (OR = 2.51; 95 % CI 0.29, 1.55) and HR-OSAS (OR = 8.24; 95 % CI 1.13, 3.09) (Table 4).

**Table 4:** Multivariate model for high risk for obstructive sleep apnea syndrome and Friedman classification 3–4.

	**HR-OSAS**				**HR-OSAS**				**Friedman classification 3–4**			
**Characteristic**	**CF**	**95% CI**	**p**	**OR**	**CF**	**95% CI**	**p**	**OR**	**CF**	**95% CI**	**p**	**OR**
**Age, 3–5 years vs 6–9 years**	0.89	−0.05, 1.83	0.06	2.37	0.69	−0.31, 1.69	0.17	1.99	0.45	−0.19, 1.08	0.17	1.56
**Sex, female vs male**	−0.03	−0.89, 0.82	0.94	1.06	0.05	−0.87, 0.97	0.92	1.05	−0.12	−0.72, 0.48	0.70	0.89
**Friedman classification, 3–4vs 0–2**	–	–	–	–	2.16	1.18, 3.13	**< .001**	8.64	–	–	–	–
**Outpatient use of antibiotics (last 12 months), yes vs no**	0.94	−0.51, 2.40	0.203	2.54	0.52	−0.99, 2.04	0.50	1.69	0.69	−0.08, 1.47	0.08	2.01
**Outpatient use of antibiotics (> 1 in the last 12 months), yes vs no**	1.71	0.68, 2.75	**0.001**	5.54	1.58	0.45, 2.72	**0.006**	4.86	0.65	−0.08, 1.40	0.08	1.92
**Hospitalizations for respiratory causes (last 12 months), yes vs no**	−0.59	−1.99, 0.81	0.41	0.55	−1.04	−2.52, 0.45	0.17	0.35	0.80	−0.06, 1.65	0.07	2.22
**Hospitalizations for respiratory causes (> 1 in the last 12 months), yes vs no**	0.96	−0.96, 2.89	0.32	2.62	1.32	−0.75, 3.37	0.21	3.71	−0.64	−2.23, 0.95	0.43	0.53
**GMFCS V, yes vs no**	1.15	0.24, 2.05	**0.01**	3.15	0.81	−0.20, 1.82	0.12	2.25	0.92	0.20, 1.55	**0.005**	2.51
**HR-OSAS, yes vs no**	–	–	–	–	–	–	–	–	2.11	1.13, 3.09	**< .001**	8.24

CF, coefficient; CI, confidence interval; GMFCS, Gross Motor Function Classification System; HR-OSAS, high risk for obstructive sleep apnea syndrome; OR, odds ratio.

## Discussion

To the best of our knowledge, this is the largest sample of children with CP ever described for OSAS assessment. The final sample was composed of children from the five regions of Brazil and 14 of the 26 states. As CP is a syndrome with a variable spectrum of severity, the sample was analyzed in its entirety (GMFCS I-V) and separately in GMFCS I-IV and GMFCS V samples.

The availability of polysomnography is quite low in the Brazilian public health system, similar to other low and middle-income countries (LMICs), where assessments from questionnaires are effective for identifying patients at increased risk for OSAS[1,17]. A study examined the association between the results of polysomnography and PSQ in 745 Turkish patients aged 2–18 years with multiple diagnoses, the CP group (80 individuals) had the highest PSQ sensitivity (88.8%) for the diagnosis of OSAS[18]. Some other studies used the PSQ [15,16] and the Sleep Disturbance Scale for Children (SDSC) in children with CP [14,19,20,22,24]. The PosaST was specifically constructed and validated for the screening of risk for OSAS. Although PSQ (22 items), SDSC (25 items), and PosaST (6 items) are validated and adapted for Brazilian Portuguese and culture, and PSQ is the most sensitive screening questionnaire for OSAS in children [25], PosaST was used in the present study because it is shorter, requiring less time and effort from parents to answer questions, making it more suitable for low-resource settings in Brazil. Furthermore, the six behavioral items of the PSQ do not allow us to differentiate whether these symptoms would be due to OSAS or primary behavioral comorbidity in children with CP, which may generate an interpretation bias when used in this population.

The main risk factor for SDB in children is adenotonsillar hypertrophy[1,2]. Respiratory illnesses are the main cause of hospital admissions [26] in CP. A consensus statement on respiratory disease in CP defined the following risk factors: GMFCS V; at least one hospital admission for respiratory illness in the past year; at least two courses of antibiotics for chest infections in the past year; dysphagia; current seizures; daily cough, or weekly wheeze, phlegm or gurgly chest; GERD; mealtime respiratory symptoms; snoring every night[27]. Based on these data, the variables to be analyzed in the present study were defined.

The prevalences of HR-OSAS were 9.0 % (GMFCS I-V) and 14.7 % (GMFCS V). In 2008 a study evaluated 998 Brazilian schoolchildren (9–14 years) via a specific questionnaire about symptoms of SDB [28], but it was not possible to use it for comparison with the present data due to the incompatible age ranges (3–9 years vs 9–14 years). Significantly higher risk factors for OSAS were found among children with CP and CP with epilepsy than among children with other developmental disabilities in 215 American children evaluated via PSQ[15]. Regarding SDB (OSAS included, but not specifically), some studies found significantly higher frequencies in children with CP (12 % to 44 %)[14,16,19,20, 21, 22,24]. SDB was significantly more prevalent in 94 Turkish children with CP aged 2–18 years in PSQ scores compared to 94 TD pairs[16]. SDB was the most common sleep disorder in 100 Egyptian children with CP evaluated via questionnaires[13]. SDB symptoms were significantly more frequent in 61 Polish children with CP than in the control group studied via questionnaires[21]. Among 173 Irish children with CP, 14.5 % experienced SDB according to SDSC[24]. Forty-one American children with CP had significantly higher SBD-PSQ scores than TD controls aged 8–12 years[19]. One hundred and nine Malaysian children with CP scored significantly higher than their healthy siblings on SDB/SDSC subscale[22]. SDB prevalences of 14.0 % and 25.6 % were found respectively in 165 Italian children with CP aged 6–16 years [14] and in 43 Brazilian children with CP aged 4–18 years [20] using the SDSC.

Studies conducted in the USA [4,5] with respectively 110 and 700 TD children evaluated through polysomnography (using AIH > 5 as the diagnostic criterion) and in China [6] with 619 TD children (using the International Criteria of Sleep Disorders version II criteria) reported OSAS prevalence rates of 1.2 % to 5.8 %. In a study analyzed via PSQ 1642 Italian TD children, 10.5 % were considered at risk for OSAS[7]. The prevalence of HR-OSAS found in the present study in the GMFCS V (14.7 %) sample was significantly higher than that reported by the studies that used polysomnography (1.2 % to 5.8 %) [4, 5, 6], but did not show a significant difference comparing with that one in which PSQ was used (10.5 %)[7]. The prevalence of HR-OSAS found in the GMFCS I-V sample (9.0 %) was not significantly higher than those of the studies that used polysomnography (1.2 % to 5.8 %) [4, 5, 6] and PSQ (10.5 %)[7].

No significant differences were found in the frequencies of age groups, sex and more than one period of antibiotic use for respiratory causes in the past year when comparing GMFCS I-IV vs GMFCS V. Contrary, Elsayed et al. (2013) found SDB significantly more frequent in school children than in preschoolers with CP[13]. The following variables were significantly more frequent in the GMFCS V than in the GMFCS I-IV sample: Friedman criteria 3–4, hospitalizations, more than one hospitalization and antibiotic use for respiratory causes in the past year, GERD, drooling and epilepsy. Based on the risk factors described above, the authors already expected to find these significant differences between the samples.

Among the confirmed hypotheses was the significantly higher prevalence of HR-OSAS in GMFCS V (14.7 %) compared to GMFCS I-IV (6.4 %). Other studies also found higher prevalences of OSAS with increasing GMFCS levels[13,15].

The multivariate model for HR-OSAS including the Friedman criteria as a dependent variable showed significant positive correlations with Friedman criteria 3–4 and more than one period of antibiotics use for respiratory causes in the last 12 months, but this model did not find a significant association with GMFCS V. Taking this finding into consideration, the authors constructed a new multivariate model for HR-OSAS excluding the Friedman criteria as a dependent variable. This new model showed significant positive associations with more than one period of antibiotics use for respiratory causes in the last 12 months and GMFCS V. Thinking about the differences found between the two models, the authors developed a multivariate model for Friedman criteria 3–4 that showed significant positive correlations with GMFCS V and HR-OSAS. Therefore, the Friedman criteria 3–4 variable had a significant correlation with HR-OSAS, regardless of GMFCS, and the GMFCS V variable became significantly associated with HR-OSAS in the GMFCS I-V sample at the expense of the significantly higher prevalence of Friedman criteria 3–4 in children classified as GMFCS V. Koyuncu E et al. (2017) also did not find significant relationships between SDB, age and sex [16]. Newman et al. (2006) did not find significant correlations between demographic and clinical variables[24].

### *Limitations and strengths*

The main limitations of this study were the convenience sample, which prevented us from generalizing these results to the population of Brazilian children with CP, and the use of PosaST which was not validated exclusively for children with CP. Therefore, the authors used as a criterion for defining HR-OSAS in children with CP the cut-off score (≥ 2.72) found in the validation study of the questionnaire in the Brazilian TD children. This cut-off score could be either higher or lower than 2.72 in children with CP, which would change the present results.

Strengths of this study include: its sample size and multicenter design, with representatives from most of the national territory; the coherence of its results showing the higher prevalence of OSAS in children with CP classified as GMFCS V in relation to TD children evaluated by polysomnography, and in children with CP with greater functional severity, and its correlation with palatine tonsils hypertrophy and more than one respiratory infection requiring antibiotic therapy in the last 12 months.

The present study suggests some possible conclusive findings. OSAS is more prevalent in children with CP classified as GMFCS V than in TD children evaluated via polysomnography. Its prevalence is higher in those with greater functional severity. More severe functional severity also determines a significantly higher prevalence of palatine tonsil hypertrophy, one or more hospitalizations, and one period of outpatient antibiotic use for respiratory causes in the last 12 months, GERD, drooling, and epilepsy. More than one period of outpatient use of antibiotics for respiratory causes in the last 12 months and palatine tonsil hypertrophy are significantly correlated with HR-OSAS. Palatine tonsil hypertrophy (Friedman 3–4) was a factor strongly and significantly associated with HR-OSAS, and its high prevalence in those classified as GMFCS V made this subsample significantly correlated with HR-OSAS. PosaST can be a reliable questionnaire in this population of patients in LMICs. Studies are needed to define the PosaST cut-off score for HR-OSAS in children with CP by comparing it with polysomnography results. It would be interesting to compare results between children with CP who have had PosaST and PSQ evaluations to analyze if one method is more accurate than the other in relation to polysomnography parameters.

## Abbreviations

AIH, apnea-hypopnea index; CI, confidence interval; CP, cerebral palsy; GERD, gastroesophageal reflux disease; GMFCS, Gross Motor Function Classification System; HR-OSAS, high risk for obstructive sleep apnea syndrome; IQR, interquartile range; LMICs, low and middle-income countries; OR, odds ratio; OSAS, obstructive sleep apnea syndrome; PosaST, Pediatric Obstructive Sleep Apnea Screening Tool; PSG, polysomnography; SD, standard deviation; SDSC, Sleep Disturbance Scale for Children; SDB, sleep-disordered breathing; SRDB, Sleep-related Breathing Disorder Scale; TD, typically developing.

## Funding source

The authors.

## Conflicts of interest

The authors have no interests which might be perceived as posing a conflict or bias.
